# Case–Control Study of Risk Factors for Acquired Hepatitis E Virus Infections in Blood Donors, United Kingdom, 2018–2019

**DOI:** 10.3201/eid2706.203964

**Published:** 2021-06

**Authors:** Iona Smith, Bengü Said, Aisling Vaughan, Becky Haywood, Samreen Ijaz, Claire Reynolds, Su Brailsford, Katherine Russell, Dilys Morgan

**Affiliations:** Public Health England, London, UK (I. Smith, B. Said, A. Vaughan, B. Haywood, S. Ijaz, C. Reynolds, S. Brailsford, K. Russell, D. Morgan);; National Institute for Health Research Health Protection Research Unit in Emerging and Zoonotic Infections, Liverpool, UK (I. Smith)

**Keywords:** HEV, hepatitis E virus, blood donation, blood-borne infections, food-borne infections, hepatitis, hepatitis E, surveillance, viral infections, viruses, zoonotic infections, zoonoses, United Kingdom, food safety

## Abstract

Hepatitis E virus (HEV) is the most common cause of acute viral hepatitis in England. Substantial yearly increases of autochthonous infections were observed during 2003–2016 and again during 2017–2019. Previous studies associated acute HEV cases with consumption of processed pork products, we investigated risk factors for autochthonous HEV infections in the blood donor population in England. Study participants were 117 HEV RNA–positive blood donors and 564 HEV RNA–negative blood donors. No persons with positive results were vegetarian; 97.4% of persons with positive results reported eating pork products. Consuming bacon (OR 3.0, 95% CI 1.7–5.5; p<0.0001), cured pork meats (OR 3.5, 95% CI 2.2–5.4; p<0.0001), and pigs’ liver (OR 2.9, 95% CI 1.0–8.3; p = 0.04) were significantly associated with HEV infection. Our findings confirm previous links to pork products and suggest that appropriate animal husbandry is essential to reduce the risk for HEV infection.

A substantial increase in locally acquired cases of hepatitis E virus (HEV) has been observed across Europe; a 10-fold increase of >21,000 cases of HEV was reported in the European Economic Area during 2005–2015 (*1*–*4*). It is difficult to accurately estimate the true burden of HEV due to substantial heterogeneity in available data across member states (*5*).

An increasing trend of acute HEV cases was observed in the United Kingdom, during 2010–2016; peaks were reported in 2015 (1,212 cases), 2016 (1,243 cases), 2018 (1,002 cases), and 2019 (1,202 cases) (*6*). HEV is the most common cause of diagnosed acute viral hepatitis in England (*2*,*7*–*9*). The annual estimate of HEV infections in England is 100,000–150,000 (*9*,*10*), and the actual burden of infection is likely to be higher. In addition to acute symptomatic infection, asymptomatic HEV infection has been reported previously (*11*) and has been observed in blood donors in the United Kingdom. The prevalence of HEV infection is dynamic in England and Wales, as suggested by the fluctuating incidence of acute HEV infections and HEV RNA presence in blood donations (*6*,*12*).

HEV is a RNA virus with 8 genotypes; genotype 1 (G1) and G2 viruses are predominantly found in low- and middle-income countries, whereas G3, G4, and G7 viruses are responsible for infections in high-income countries (*13*). G1 and G2 are transmitted by the fecal–oral route; infection with G3 and G4 viruses is primarily foodborne. HEV is found in many animal species; however, pigs are recognized as the main reservoir (*14*,*15*). A high prevalence of antibodies to HEV in UK swine has been reported (92.8%), along with evidence of current HEV infection in 20.5% (95% CI 17.2%–23.8%) of pigs at the time of slaughter (*16*). These findings were determined using HEV RNA detection in either plasma or cecal samples; HEV was detected in 22/629 (3.5%) of plasma samples and 93/629 (14.9%) of cecal contents (*16*–*18*). The presence of HEV RNA in cecal samples could be caused by environmental contamination; however, multiple other studies in Europe have also observed the presence of HEV RNA at the point of slaughter (*19*–*21*). This presence of viremia at time of slaughter poses a significant risk for HEV-infected products to enter the food chain.

In general, in the United Kingdom, G3 clade 1 (G3 efg) viruses circulate in swine; however, the increase of acute HEV cases in England in 2010 coincided with the emergence of a novel HEV phylotype, G3 clade 2 (G3 abcdhij) viruses (*22*). No evidence has been found of this phylotype in the pig population in England and, although it has been isolated in 1 pig in Scotland, the isolate fell outside of the dominant human clade (*16*). It is likely, therefore, that the reason the novel phylotype is present is the consumption of pork originating from outside the United Kingdom (*3*,*22*–*24*). Viruses detected in human clinical samples in the United Kingdom, which are closely related to those found in pigs in mainland Europe (*3*,*22*) support this idea; taken together, the evidence suggests a risk for zoonotic transmission from pork products originating from outside of the United Kingdom.

A body of evidence supports the finding that HEV infection can also be acquired from blood products and HEV can be transmitted through transfusion (*25*), and the clinical consequences have been increasingly recognized (*26*,*27*). Therefore, to mitigate the risk for transmission by transfusion, National Health Service Blood and Transplant (NHSBT) introduced HEV-screened components for selected patients in March 2016 following a recommendation from the Advisory Committee on the Safety of Blood, Tissues and Organs (SaBTO). SaBTO subsequently recommended that the UK blood services implement universal screening; beginning April 2017, all blood components have been screened and those used are HEV negative (*28*,*29*).

In addition to mitigating the risk for transmission via transfusion, universal screening of blood donations by NHSBT also provided a new and unique opportunity to understand HEV infection in a population that more closely reflects the general population. The aim of this study was to characterize the clinical features of UK-acquired HEV infection in blood donors in England and investigate the potential risk factors for infection.

## Methods

### Study Design

We conducted a case–control study April 1, 2018–March 31, 2019, to describe the clinical features of infection and to identify the risk factors for HEV acquisition within the blood donor population in England. As part of the continuing enhanced surveillance, NHSBT contacted all blood donor HEV cases to inform them of their infection. HEV RNA positive samples, detectable by PCR, were sent to the Blood Borne Virus Unit, Public Health England (Colindale, UK) for genotyping (*26*). NHSBT invited donors to take part in the case–control study, which included a link to complete an enhanced surveillance questionnaire designed for this study (*29*). All participants provided consent for their information to be used in the study.

We used case age and donation date to request a sample of eligible controls from NHSBT Donor Insight; they created a dispatch extract of data from their donor database in Excel (Microsoft, https:///www.microsoft.com) containing donor’s name, postal address, email address, donor number, and donation date. No PII about the controls was shared outside of NHSBT. Controls were age-matched to cases across defined age groups (17–24, 25–44, and >45 years) and had donated within the same week as the age-matched case. Controls were not sex-matched to cases; we adjusted for sex in the analysis.

### Case Selection

A case was defined as a blood donor, residing in England, who donated blood to NHSBT during the period April 1, 2018–March 31, 2019; who was HEV RNA positive as indicated by a confirmed positive HEV RNA donation testing result; and who had no history of travel outside the United Kingdom in the 9 weeks before donation. NHSBT collected descriptive data for all HEV RNA–positive donors identified within the study period to ensure the cases included in the study were representative of the HEV RNA positive blood donor group.

### Control Selection

A control was defined as a blood donor who contemporaneously donated blood to the NHSBT, resides in England, and was confirmed negative for HEV and all other markers of infection during screening. They also had no travel history outside the United Kingdom in the 9 weeks before donation, had not been recently surveyed by NHSBT, and had not opted out of communications from NHSBT.

### Data Collection

To characterize the clinical features of indigenously acquired HEV infection and risk factors for HEV infection, we collected the following information from cases and controls: travel history, animal exposures, environmental exposures, alcohol intake, medication, and concurrent conditions. We also asked about the food they consumed and their purchasing preferences; on the basis of published evidence, we included more detailed questions about the consumption of pork products or derivatives. Because of the long incubation period of HEV (2–9 weeks), questions were phrased as, “Are you likely to have eaten the following food items?” We asked cases about the 9-week period before the date of their HEV RNA positive blood donation and asked controls about the 9-week period before their donation.

Study participants who reported a travel history outside the United Kingdom in the 9 weeks (the maximum incubation period for HEV) before donating were excluded from the analysis because they could have contracted HEV through their travel (*30*). In terms of patient-identifiable information (PII), controls were not asked to provide any, and their survey data were not linked to their donation record. Cases were requested to provide their name to allow for linkage of questionnaire responses with their laboratory results. Participants were excluded if the questionnaire was incomplete.

Ethics approval was not required because this study used data that were routinely being collected through enhanced surveillance. All participants, however, did consent for their information to be used in the case-control study. PII was removed before analysis, and all data were handled according to Caldicott principles. 

Of 411 participants identified as HEV RNA–positive blood donors, 182 (44%) completed the questionnaire. Compared to other studies using online surveys with a blood donor population, which have reported 26% completion of questionnaires, 44% is a strong response rate (*31*). After we excluded duplicate questionnaires, participants who had traveled in the designated period, and those with missing information, 117 HEV RNA–positive blood donors remained in the study.

### Data Analysis

To investigate potential risk factors and environmental exposures of HEV, we conducted univariate logistic regression. We included variables with an odds ratio (OR) >1 and p<0.01 in a multivariable logistic regression model. In the multivariable analysis, variables with an adjusted OR <1 or no significant association (p>0.05) with HEV infection were removed from the model in a backward stepwise fashion until all the variables in the model exhibited a degree of association. We adjusted for age and sex at all stages of analysis. We performed all statistical analyses using Stata software version 15 (StataCorp, https://www.stata.com).

## Results

### Study Demographics

We included a total of 117 HEV RNA–infected blood donors and 564 HEV RNA–negative donors in the case–control study (Figure). The ratio of cases to controls was 1:4.8. The HEV RNA–infected participants corresponded to 28.5% (117/411) of the total infected blood donors identified during the study period (Table 1). As in previous studies (*9*,*26*,*29*), most of the HEV-infected blood donors identified in the study period were male, a similar proportion to the HEV-infected blood donors included in the study. Donors in the age group >45 years were the greatest number of all HEV-infected and noninfected blood donors; the predominance of this age group was seen in cases and controls in the study.

**Figure:**
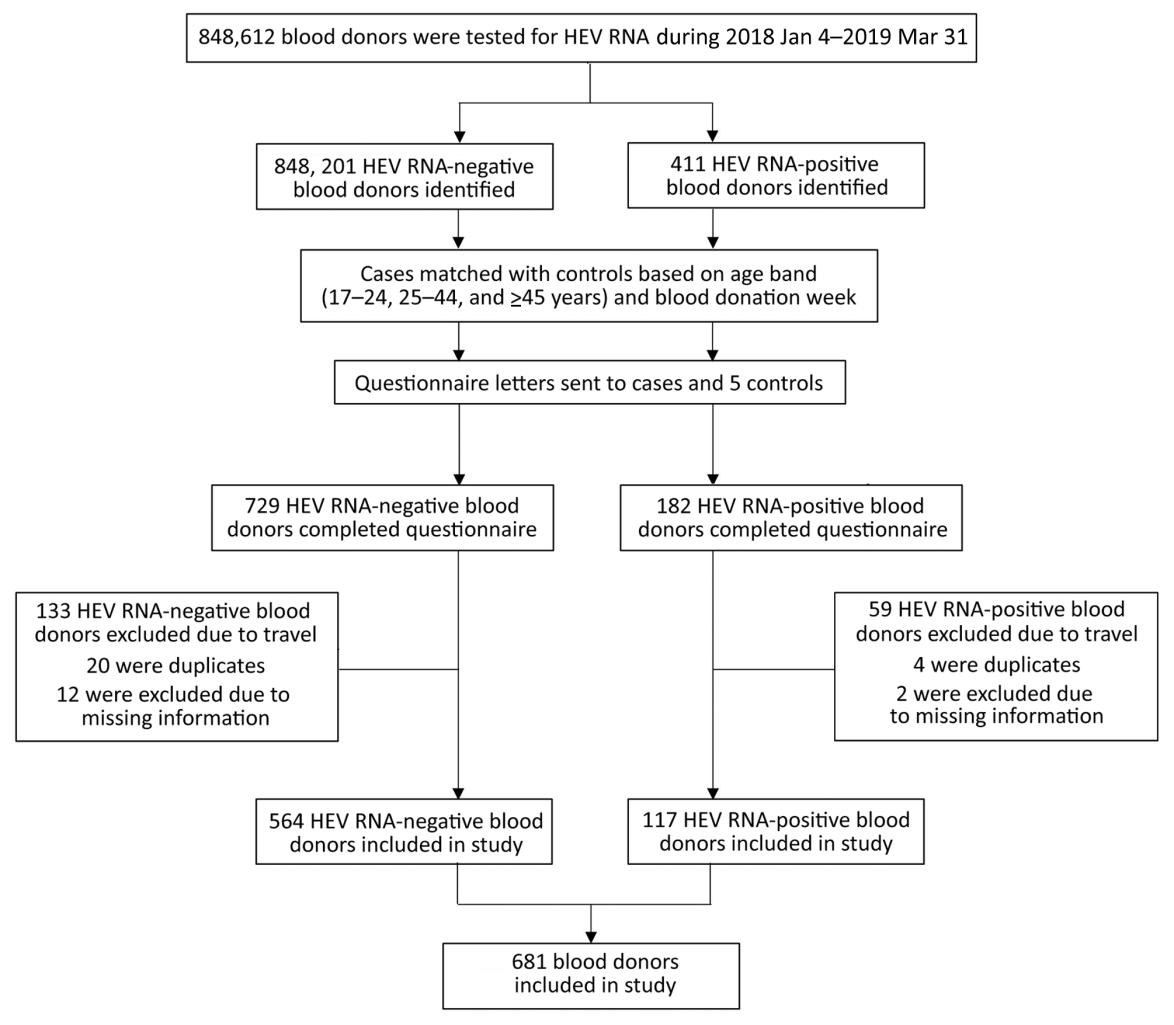
Recruitment of blood donors to case–control study of hepatitis E virus in blood donors, United Kingdom, 2018–2019.

**Table 1:** Age and sex of participants in case–control study of hepatitis E infections in blood donors, 2018–2019*

Characteristic	All HEV RNA–positive samples, n = 411	All HEV RNA–negative samples, n = 848,201	Cases, n = 117	Controls, n = 564
Age group, y				
17–24	24 (5.8)	87,460 (10.3)	5 (4.3)	15 (2.7)
25–44	159 (38.7)	337,240 (39.8)	31 (26.5)	184 (32.6)
>45	228 (55.5)	423,501 (49.9)	81 (69.8)	365 (64.7)
Sex				
F	165 (40.2)	486,388 (57.3)	52 (44.4)	305 (54.1)
Median age, y	45	42	48	50
Age range, y	17–80	17–95	20–78	18–78
M	246 (59.9)	361,813 (42.7)	65 (55.6)	259 (45.9)
Median age, y	49	48	54	52
Age range, y	18–73	17–84	21–72	17–81

*Values are no. (%) participants except as indicated.

### HEV Genotype

We conducted sequence and phylogenetic analysis on 24 (20.5%) HEV RNA–positive cases, where viral load was sufficient to do so (*32*,*33*). Phylogenetic analysis showed that all viruses belonged to the HEV G3 phylogroup; 22 (91.7%) of them were HEV G3 clade 2 (abcdhij) viruses and 2 (8.3%) were G3 clade 1 (efg) HEV viruses.

### Symptoms

Overall, 41/117 (35%) of cases were symptomatic, and female and male cases experienced symptoms equally. The most commonly reported symptoms were fatigue, joint pain, and headaches; these symptoms were reported by 14%–20% of those with symptoms. Other symptoms experienced by ≈10% of cases included abdominal pain, nausea, change in appetite, and weakness or tingling. All symptoms were experienced significantly more by cases than controls except vomiting, which 1 case and no control reported (p<0.0001). Overall, 76/117 (65%) of cases and 552/564 (98%) of controls were asymptomatic.

### Risk Factors

In the univariate analysis of 19 food items that were likely to have been consumed over the 9-week period before onset of symptoms in the cases and the previous 9-week period to donation in controls, 14 food items were significantly associated with HEV infection (OR >1; p<0.01). Most of these items were animal products (Table 2). No cases and 4 controls were vegetarians. Contact with animals, specifically dogs, was associated with HEV infection (OR 1.7, 95% CI 1.1–2.6; p = 0.01); however, upon inclusion in multivariable analysis, this factor lost significance. The final multivariable model, which was adjusted for age and sex, showed that the only variables of note were bacon, cured pork meats such as sliced salami and cabanos, and pigs’ liver (Table 3). 

**Table 2:** Univariate analysis of risk factors for hepatitis E infection in study of blood donor–related transmission, 2018–2019*

Risk factor	Cases, n = 117	Controls, n = 564	Univariable analysis		
			OR ((95% CI)		p value
Food consumption					
Bacon‡	102 (87.2)	343 (60.8)	4.6 (2.6–8.2)		<0.0001
Cured pork meat‡	73 (62.4)	158 (28.0)	4.5 (3.0–6.9)		<0.0001
Ham (off-the bone or joint)	42 (35.9)	148 (26.2)	1.6 (1.0–2.4)		0.04
Other pork products	26 (22.2)	79 (14.0)	1.7 (1.0–2.8)		0.04
Other sausages	11 (9.4)	59 (10.5)	0.9 (0.4–1.7)		0.7
Pate‡	35 (29.9)	83 (14.7)	2.5 (1.6–3.9)		<0.0001
Pigs’ liver‡	8 (6.8)	10 (1.8)	3.7 (1.4–9.8)		0.01
Pork‡	72 (61.5)	269 (47.7)	1.8 (1.2–2.6)		0.01
Pork pie	40 (34.2)	142 (25.2)	1.5 (0.9–2.2)		0.09
Pork sausages‡	95 (81.2)	340 (60.3)	2.9 (1.7–4.7)		<0.0001
Sliced sandwich ham, prepacked‡	81 (69.2)	307 (54.4)	1.9 (1.3 – 3.0)		0.003
Any pork product‡	114 (97.4)	445 (78.9)	10.5 (3.3–33.6)		<0.0001
Chicken	107 (91.5)	443 (78.6)	3.0 (1.5–5.9)		0.002
Fish	84 (71.8)	395 (70.0)	1.1 (0.7–1.7)		0.7
Game	9 (7.7)	18 (3.2)	2.4 (1.1–5.5)		0.04
Other offal‡	16 (13.7)	34 (6.0)	2.3 (1.2–4.3)		0.01
Shellfish	44 (37.6)	191 (33.9)	1.3 (0.8–1.9)		0.3
Fresh fruit‡	109 (93.2)	472 (83.7)	2.8 (1.3 – 6.0)		0.01
Raw vegetables	81 (69.2)	354 (62.8)	1.5 (1.0–2.3)		0.08
Salad vegetables‡	108 (92.3)	460 (81.6)	3.0 (1.5–6.2)		0.01
Supermarket					
Supermarket A‡	60 (51.3)	202 (35.8)	2.0 (1.3 – 3.0)		0.001
Supermarket B	33 (28.2)	159 (28.2)	1.0 (0.7–1.6)		0.9
Supermarket C	71 (60.7)	312 (55.3)	1.2 (0.8–1.9)		0.3
Supermarket D	15 (12.8)	114 (20.2)	0.6 (0.3–1.0)		0.05
Supermarket E	58 (49.6)	245 (43.4)	1.3 (0.8–1.9)		0.3
Supermarket F	40 (34.2)	150 (26.6)	1.4 (0.9–2.1)		0.1
Supermarket G‡	40 (34.2)	123 (21.8)	1.8 (1.2–2.8)		0.01
Supermarket H	20 (17.1)	112 (19.9)	0.8 (0.5–1.4)		0.5
Supermarket I‡	33 (38.2)	112 (19.9)	1.6 (1.0–2.5)		0.04
Supermarket J	0 (0.0)	2 (0.4)	1 (NA)		NA
Supermarket K	1 (0.9)	14 (2.5)	0.4 (0.1–2.8)		0.3
Local butcher/shop	17 (7.7)	49 (2.3)	3.6 (1.5–8.8)		0.0
Animal contact					
Yes‡	97 (82.9)	389 (69.0)	2.3 (1.4–3.9)		0.0
Cat	48 (41.0)	209 (37.1)	1.2 (0.8–1.8)		0.4
Dog‡	76 (65.0)	297 (52.7)	1.7 (1.1–2.6)		0.01
Rodent	8 (6.8)	24 (4.3)	1.7 (0.8–4.0)		0.2
Pig	5 (4.3)	15 (2.7)	1.6 (0.6–4.5)		0.4
Sheep	2 (1.7)	29 (5.1)	0.3 (0.1–1.4)		0.1
Horse	6 (5.1)	39 (6.9)	0.8 (0.3–1.8)		0.5
Cow	3 (2.6)	20 (3.6)	0.7 (0.2–2.4)		0.6
Alcohol consumption†					
Yes‡	95 (81.2)	395 (70.0)	1.8 (1.1–3.0)		0.02
1–10 units/wk‡	66 (57.4)	279 (49.8)	1.9 (1.1–3.1)		0.02
10–20 units/wk	17 (14.8)	71 (12.7)	1.8 (0.9–3.5)		0.1
>20 units/wk	10 (8.7)	41 (7.3)	1.6 (0.7–3.7)		0.3
Underlying illnesses					
Medical condition	30 (25.6)	130 (23.1)	1.1 (0.7–1.8)		0.7
Respiratory	4 (3.4)	19 (3.4)	1.1 (0.4–3.2)		0.9
Liver	1 (0.9)	2 (0.4)	1.9 (0.2–21.4)		0.6
Heart	0 (0.0)	1 (0.2)	1 (–)		–
Diabetes	3 (2.6)	5 (0.9)	2.4 (0.6–10.5)		0.2

*Values are no. (%) participants except as indicated. OR, odds ratio. (†Missing information for 2 cases. (‡Significant at univariate analysis.

**Table 3:** Multivariable analysis model of food consumption associated with testing positive for hepatitis E virus, adjusted for age and sex

Risk factor	Multivariable analysis	
	OR (95% CI)	p value
Bacon		<0.0001
No	Referent	
Yes	3.0 (1.7–5.5)	
Cured pork meat		<0.0001
No	Referent	
Yes	3.5 (2.2–5.4)	
Pigs’ liver		0.04
No	Referent	
Yes	2.9 (1.0–8.3)	

## Discussion

The overall prevalence of HEV infection in blood donors detected during this study period is 0.05% (411/848,201). Although we found a higher level of infection than reported previously (*29*), the rate does fluctuate; the study rate shows the continued presence of HEV in the blood donor population, indicating the importance of blood screening. Compared with previous populations used for investigating HEV, the blood donor population is more representative of the general population and has different demographics than the population of acute HEV cases.

We observed a greater presence of HEV in male than female blood donors. This difference between sexes has been a consistent finding in previous studies in England and across Europe (*4*,*11*,*22*,*29*,*34*–*36*). One explanation for the dominance of male cases could be a difference in the consumption of meat (*22*). In the United Kingdom, the National Diet and Nutrition Survey collects information about the diet, nutrient intake, and the nutritional status of the UK population. Their data show that men consistently consume more meat than women (*37*). An alternative explanation for the sex difference is that men may be more clinically susceptible because of sex-driven differences, whereas women are less likely to exhibit acute clinical disease (*30*). However, this study found that symptoms were experienced equally by male and female cases. Ijaz et al. previously noted that men and women have similar levels of IgG for HEV in England (*10*), which suggests a comparable burden of HEV in both sexes. Similar sex differences have also been observed with other hepatitis viruses (*38*,*39*). The reason why more HEV cases are male remains uncertain and warrants further research.

Previous studies have suggested that the distribution of HEV cases is not uniform and that certain areas in England are at greater risk (*4*,*30*,*40*). The results of our case–control study, however, did not identify geographic clustering of HEV cases among blood donors; this was consistent with the findings of a NHSBT surveillance study of HEV-positive blood donors during March 2016–December 2017 (*29*). As observed in the NHSBT surveillance study, HEV G3 clade 2 was the predominant phylotype detected (*4*,*29*), which suggested that the HEV infections identified in the blood donor population may have resulted from consuming pork products that originated from outside the United Kingdom, because HEV G3 clade 2 is not the predominant phylotype circulating in pigs (*3*,*4*,*16*). Determining the virus’s origin is difficult, however, because it is generally accepted that HEV is endemic in swine within the United Kingdom and mainland Europe (*16*,*19*–*21*,*41*,*42*).

Ours is not the first UK case–control study to find that processed pork products and pigs’ liver are associated with HEV infection (*30*,*43*,*44*). However, it is one of the first to identify bacon and cured pork meat as risk factors for HEV. The relative contribution of each pork product to the total number of cases should be noted, though; preventing the consumption of pigs’ liver would lead to only a modest reduction in HEV cases because relatively few persons eat the liver. Unlike previous studies, this study did not find risk associated with the consumption of pork pies or the consumption of ham and sausages purchased from a specific UK supermarket chain (*30*). Possible causes are changes in the supplier or source of pork for the supermarket chain since the findings of the previous study. Alternatively, there may be differences between the study populations.

Recent studies have investigated the thermal stability of HEV; researchers have not agreed upon the necessary time and temperature for heat inactivation of HEV (*30*,*45*–*47*). Although bacon should be cooked, other cured meats, such as sliced salami and cabanos, may not have been cooked during the curing process, and it is currently unknown whether curing is sufficient to inactivate HEV (*48*). HEV contamination has previously been found in raw meat products (*17*,*18*,*49*); thus, it is biologically feasible that if the curing process was not sufficient to inactivate the HEV then viral transmission could occur from consumption of these products. Further studies are required to understand the parameters required for heat inactivation of HEV and the effect of different treatment procedures such as curing on the virus. Unfortunately, methods for sampling and testing of pork and other food products are not sufficiently robust to provide information about contamination with infectious viruses.

A limitation of this study is that some blood donors may have previously been infected with HEV and so were not at risk for infection at the time of study. Testing HEV RNA–negative persons for HEV antibodies would have clarified this, but that was not possible in this study.

Of note is the potential effect of recall bias for study participants recounting their potential food and environmental exposures. HEV RNA–positive blood donors were contacted as soon as possible after the donation was confirmed HEV RNA positive. However, the maximum 9-week incubation period of HEV may have led to patients forgetting their food and environmental exposures or recalling them incorrectly. Furthermore, controls would have had a larger time lag due to the time required to identify appropriate controls based on case demographics and to send out the appropriate information. The lag could increase the likelihood of recall bias. In addition, sharing with study participants the information about hepatitis E and its association with pork may have biased the participants’ recall response.

Our knowledge of HEV infection in the population was previously limited to a population of acutely infected persons who sought medical care. The introduction of universal screening has led to the availability of an immensely useful cohort of HEV-infected persons different from the cohort of acute HEV cases. HEV-infected blood donors were identified not through medical investigations but through universal screening; thus, they are more representative of the general population compared with the acute HEV population. However, we recommend caution before extrapolating the results of this study to the general population. Because of donor selection guidelines (*50*), the donor population tends to be healthier than the general population; the cutoff of 65 years in new donors and the self-selecting nature of donation suggests that the prevalence of HEV in the general population is different than that found in the blood donor population.

This study found that HEV infection in blood donors in England was associated with the consumption of 3 pork products; bacon, cured pork meats, and pigs’ liver. Bacon and other cured pork meats were not previously identified as risk factors for HEV. The identification of these pork products highlights the importance of accurate information about cooking requirements as well as the role and importance of animal husbandry to prevent HEV infection in pigs. Targeting HEV infection at the source would prevent foodborne transmission to the population.
